# Incidence of prostate cancer in Eritrea: Data from the National Health Laboratory, Orotta Referral Hospital and Sembel Hospital 2011-2018

**DOI:** 10.1371/journal.pone.0232091

**Published:** 2020-04-23

**Authors:** Lidia Biniam Medhin, Oliver Okoth Achila, Biniam Efrem Syum, Kibrom Hailu Gebremichael, Salih Mohammed Said, Hartmut Lobeck, Yosief Tewolde Ghidei

**Affiliations:** 1 Pathology, National Health Laboratory, Asmara, Eritrea; 2 Clinical Laboratory Sciences, Orotta School of Medicine and Health Sciences, Asmara, Eritrea; 3 Microbiology, National Health Laboratory, Asmara, Eritrea; 4 Surgical department, Orotta Referral Hospital, Asmara, Eritrea; Morehouse School of Medicine, UNITED STATES

## Abstract

**Introduction:**

Up-to-date statistics on prostate cancer incidence and causative risk factors are essential for the primary prevention of this disease. However, the incidence of Prostate cancer (ICD-10 code C61) (PCa), or cancers in general, are poorly documented in Eritrea. This study analyses the data available to produce an estimate of the incidence of PCa in Eritrea.

**Methods:**

We conducted a retrospective study by identifying all incident cases of PCa captured between 2011–2018 in the National Health Laboratory pathology database (Polytech 8.37.C); Urology departments of Orotta Referral Hospital and Sembel Hospital. Crude incidence rates (CIRs), age-adjusted rates per 100,000 person years and associated trends were subsequently calculated. Joinpoint Regression Program, V.4.5.0.1 was employed in these analyses.

**Results:**

A total of 1721 cases were reported, of which 1593 (92.5%) were benign prostatic hypertrophy cases and 128 (7.5%) were PCa cases. The mean (±SD) age of the patients with PCa was 73.49 (± 8.9), confidence interval (CI) (54–98) and the minimum and maximum ages were 54 and 98, respectively. The median age interquartile ranges (IQR) was 73 (13) years. The highest and lowest PCa incidence rates were in 2017 (4.51 per 100 000) and 2014 (2.69 per 100 000), respectively. The age standardised rates (ASIR) (World) over the study period (2011–2018) was 30.26 per 100 000. The annualized ASIR values over the study period was 3.78 per 100 000. The associated average annual percentage change (APC) (CI) over the study period was 5.4 (-1.4–12.7), P-value = 0.100, showing a static trend over the study period.

**Conclusion:**

This study suggests that previous reports have under-estimated the incidence of PCa in Eritrea. The study provides ample evidence on the need for research targeted at uncovering the true burden of PCa in Eritrea. Potential solutions will require the establishment of high-quality population-based cancer registries (PBCRs) and long-term commitment to improvements in research, training, screening, diagnosis, and the overall management of PCa in the country.

## Introduction

Prostate cancer (ICD-10 code C61) (PCa) is currently rated as one of the most common malignancies among men and the second leading cause of death in both high/very-high and low/medium human development index (HDI) countries, after lung and bronchus cancers, among men worldwide [1, 2, 3]. According to GLOBOCAN 2018 [3], the International Agency for Research on Cancer ‘s online database, the global age standardized rate (ASR) for PCa was 29.3 and estimated 358 989 deaths per year making it the most frequently diagnosed malignancy among men in 105 out of 185 countries [3]. Similar to previous reports, the report indicated that PCa incidence varies by a factor of more than 25 worldwide; the rates are highest in some developed countries like the United State (US) (109.2 per 100 000 age adjusted to the 2000 US standard population) and an estimated death rate of 19.2 per 100 000 [2]. In the Asia Pacific region, a recent report intimated that the ASR are expected to almost double by 2030 [4].

In Africa, a recent systematic and meta-analytical review reported a continent-wide pooled incidence rate of 22.0 (95% CI: 19.93–23.97) per 100 000 population [5]. The average risk that a man in sub-Saharan Africa (SSA) will develop prostate cancer before the age of 75 years is 3.4% [6]. Thus, the disease affects almost 1 in 30 men in the region–a risk estimate that is equivalent to that of cervical cancer (3.8%) and breast cancer (3.5%) [6]. Further, previous data from Institute for Health Metrics and Evaluation (IHME) estimated that disability adjusted life years (DALYs) from PCa increased from 100,200 in 1990 to 219,700 in 2010, and deaths also increased from 5,600 to 12,300 over the same period [6]. Boccon-Gibod’s “pussycat” (indolent tumors) and “tigers” (aggressive tumors) conundrum [7] notwithstanding; the possibility that the disease is largely underdiagnosed in Africa is substantial [8, 9].

Further, and as previously noted, there is a widespread consensus that the higher incidence of PCa is statistically biased–higher in developed countries. This outcome is largely attributed to the ready availability of prostate specific antigen (PSA) testing (an early detection system) or digital rectal exam (DRE) or other more advanced tests and the greater accuracy of national cancer registration systems (NCRS) in developed countries [9, 10]. Although PSA testing has a greater impact on the geographical variability of incidence rates *vis-à-vis* mortality; mortality-to-incidence rate ratio (MR/IR) are relatively higher for low- and medium-income countries (LMIC).

Despite this seemingly disproportionate impact of PCa in sub-Saharan Africa (SSA), it remains a low priority on the healthcare agendas in the region. Predictably, little data exist on the incidence or mortality rates of PCa in SSA [5]. According to some authors, shortage of trained health staffs (including oncologists, urologists, pathologists, radiologists), weak health management information systems and poor routine record-keeping in most health facilities in Africa continue to undermine research output on cancer and public health interventions [5, 11]. To highlight the gravity of this problem, GLOBOCAN data on cancers (including PCa) for many countries in SSA are routinely corrected for under-reporting/under ascertainment or incompleteness and inaccuracies of data [12, 10]. This is usually accomplished by extrapolating data from a contextually similar country with relatively well-equipped and regularly updated population-based cancer registry [12].

In aggregate, incidence studies are an important part of cancer monitoring within populations. Moreover, incidence information may assist in the quantification of PCa burden and may act as a crude indicator for shifting patterns of risk indicators or adverse outcomes. This study was therefore designed to provide information on the incidence of PCa in Eritrea. The study also identifies gaps in the epidemiological data which need to be filled to give a more comprehensive picture of the incidence of PCa in the country.

## Method and materials

### Study design and setting

We conducted a retrospective study by identifying all incident cases of PCa captured between 2011–2018 National Health Laboratory (NHL) pathology database (Polytech 8.37.C), urology departments of Orotta Referral Hospital and Sembel Reference Hospital. Currently, NHL, located in Asmara, the capital city of Eritrea, serves as a referral facility for all hospitals in Eritrea and has the only histopathology and cytology laboratory in the country. Therefore, it can be presumed that all histopathological specimens from patients in Eritrea were processed at the facility. Between 2011–2018, the facility received 10053 biopsy specimens (158 were prostrate biopsies). The two institutions also serve as referral facilities for patients in the country and provide specialized medical services such as computed tomography (CT), magnetic resonance imaging (MRI) and surgical services to referred patients (note that these advanced imaging techniques—CT and MRI, are only available in this facilities). The three facilities lie at the apex of an otherwise straight-forward/robust referral network and can thus provide fairly reliable information on PCa incidence. A master data set compiled from the three repositories was generated, cleaned and subsequently cross-checked for duplicate entries. Information recorded in each case includes name, date of birth or age, incident date, basis of diagnosis, area of residents. Histopathological characteristic including morphology, behavior, differentiation status and stage (number staging), among others was extracted from NHL database. Tumor classification was based on International Classification of Diseases (ICD) for Oncology, 3^rd^ ed. (ICD-10 and ICD-O) [13]. Tumor grade was evaluated using the Gleason score [14].

### Case definition and ascertainment

The case definition for inclusion included diagnosis of PCa confirmed by a recognized medical practitioner or other benign tumour processed at the facility over the specified period. Only specimen from local residents were considered for analysis. As previously noted, case finding, abstraction, and coding was undertaken using a multisource approach which included data from the pathology database at NHL and information from urology departments of two specialized referral facilities.

### Completeness and data quality

In evaluating cancer incidence, ensuring that incident cases in the population are included is critical. In this study, we evaluated completeness using a number of strategies including stability of incidence rates over time. The use of mortality-to-incidence ratios was not possible due to lack of data on PCa–related mortalities. Age-specific incidence curves were subsequently evaluated for atypical fluctuations, such as an unexpected drop in the rate of increase in incidence in older age groups. The latter outcomes may be indicative of underascertainment within these groups (although other explanations may suffice). Residency can also reveal problems with the source files, particularly under represented regions of the country. Proportion of microscopically verified specimen (MV%) was also used as a measure of data validity.

### Data analysis

Anonymised data from each repository were aggregated into a master data set (Microsoft Excel Sheet) for subsequent analysis. Crude incidence rate (CIR), was calculated by dividing the total number of cases by the corresponding population at risk [15], expressed per 100 000 person-years. Age-adjusted rates per 100,000 person years were calculated by using the direct method using 5 -year age bands (≤ 55, 55–59, 60–64, 65–69, 70–74, 75–79, 80–84 and ≥ 85. The hypothetical World population [16] was used as the standard population in this analysis. We quantified the trends using Joinpoint Regression Program V.4.5.0.1 [17]. In this analysis, the annual percentage change (APC) in rates between trend-change points (e.g. Joinpoint segment) was calculated. The average annual percentage change (AAPC), estimated as a weighted average of the estimated APC in each joinpoint segment, was subsequently used to summarize the trends in each analysis. The 95% confidence intervals (CIs) were obtained with standard error from the fit of the regression and the t-distribution function.

### Ethical approval and consent

The study was approved by the Health Research Ethics and Protocol Review Committee of the Eritrean Ministry of Health (MOH). Consent to participate was not obtained from the patients. This waiver was premised on the consideration that the study was based on anonymized patient’s records. And the data collection was done between October 2019-December 2019.

## Results

### Characteristics of participants

A total of 1721 cases were reported, of which 1593 (92.5%) were BPH cases and 128 (7.5%) were PCa cases. The mean (±SD) age of the patients with PCa was 73.49 (± 8.9) confidence interval (CI) (54–98) and the minimum and maximum ages were 54 and 98, respectively. The median age (interquartile range (IQR)) was 73 (13) years. The number of patients from the disparate administrative zones is shown in Table 1. A large proportion of the patients were from Maekel 89, (69.85%) and the lowest number of patients were from Southern and Northern Red Sea.

**Table 1:** Cases from the disparate administrative Zones in Eritrea (2011–2018).

Administrative Regions	Benign N (%)	Malignant N (%)	Total (N (%)
Maekel	1029 (64.6)	89 (70)	1118 (65.0)
Anseba	76 (4.8)	7 (5)	83 (5)
Gash Barka	109 (6.8)	10 (8)	119 (7)
Debub	317 (19.9)	17 (13)	334 (19)
Northern Red Sea	46 (2.9)	4 (3)	50 (3)
Southern Red Sea	16 (1.0)	1 (1	17 (1.0)
**Total**	**1593 (92.5)**	**128 (7.43)**	**1721 (100.0)**

Between 2011 and 2018, 10053 biopsy samples were processed at the NHL’s pathology department. The total number of prostate biopsy specimen was 158. Table 2 shows the characteristics of the tumors in terms of tumor type, differentiation and grade (Gleason Score).

**Table 2:** Characteristics of microscopically verified prostate malignancies (2011–2018).

Tumour characteristic	Patient number N (%)
**Types of Tumors**	
**Malignant**	47 (30.72)
**Benign**	106 (69.28)
**Malignant Tumors**	
** Differentiation**	
**Adenocarcinoma**	46 (97.9)
**Squamous cell Carcinoma**	1 (2.1)
**Grade (Gleasson Score)**	
**Grade 1 (Gleason score ≤ 6)**	6 (12.8)
**Grade 2 (Gleason score 3 + 4 = 7)**	8 (17.0)
**Grade 3 (Gleason score 4 + 3 = 7)**	12(25.5)
**Grade 4 (Gleason score 8)**	7 (14.9)
**Grade 5 (Gleason score 9–10)**	14 (29.8)

Disaggregation of the data sets used in this analysis according to the previously highlighted age bands shows that the largest proportion of the cases were in the 70–74 age band. The lowest proportion were in the < 50 years age band. Table 3. In a separate analysis, the data shows that age-specific rates of PCa were: 0.8%, ages < 55 years; 2.3%, ages 55–59 years; 13.3% ages 60–64; 18.8% ages 65–69 years; 21.1% ages 70–74 years; 15.6% ages 75–79 years, 15.6% ages 80–84 years and 12.5 ages ≥ 85 years.

**Table 3:** Number of prostate cancer and benign prostatic hypertrophy reported to the National Health Laboratory (NHL) pathology database, urology departments of Orotta Referral Hospital and Sembel Hospital between 2011–2018.

Condition	Age in Years N (%)								
	< 55	55–59	60–64	65–69	70–74	75–79	80–84	≥85	Total
**PCa**	1 (0.8)	3 (2.3)	17 (13.3)	24 (18.8)	27 (21.1)	20 (15.6)	20 (15.6)	16 (12.5)	128
**BPH**	50 (3.1)	64 (4.0)	228 (14.3)	293 (18.4)	356(22.3)	292 (18.3)	190 (11.9)	120 (7.5)	1593
**Total**	51 (3)	67 (3.9)	245 (14.2)	317(18.4)	356(22.3)	312 (18.1)	210(12.2)	136(7.9)	1721

**BPH**: Benign Prostatic Hypertrophy, **PCa**: Prostate cancer.

**Fig 1:**
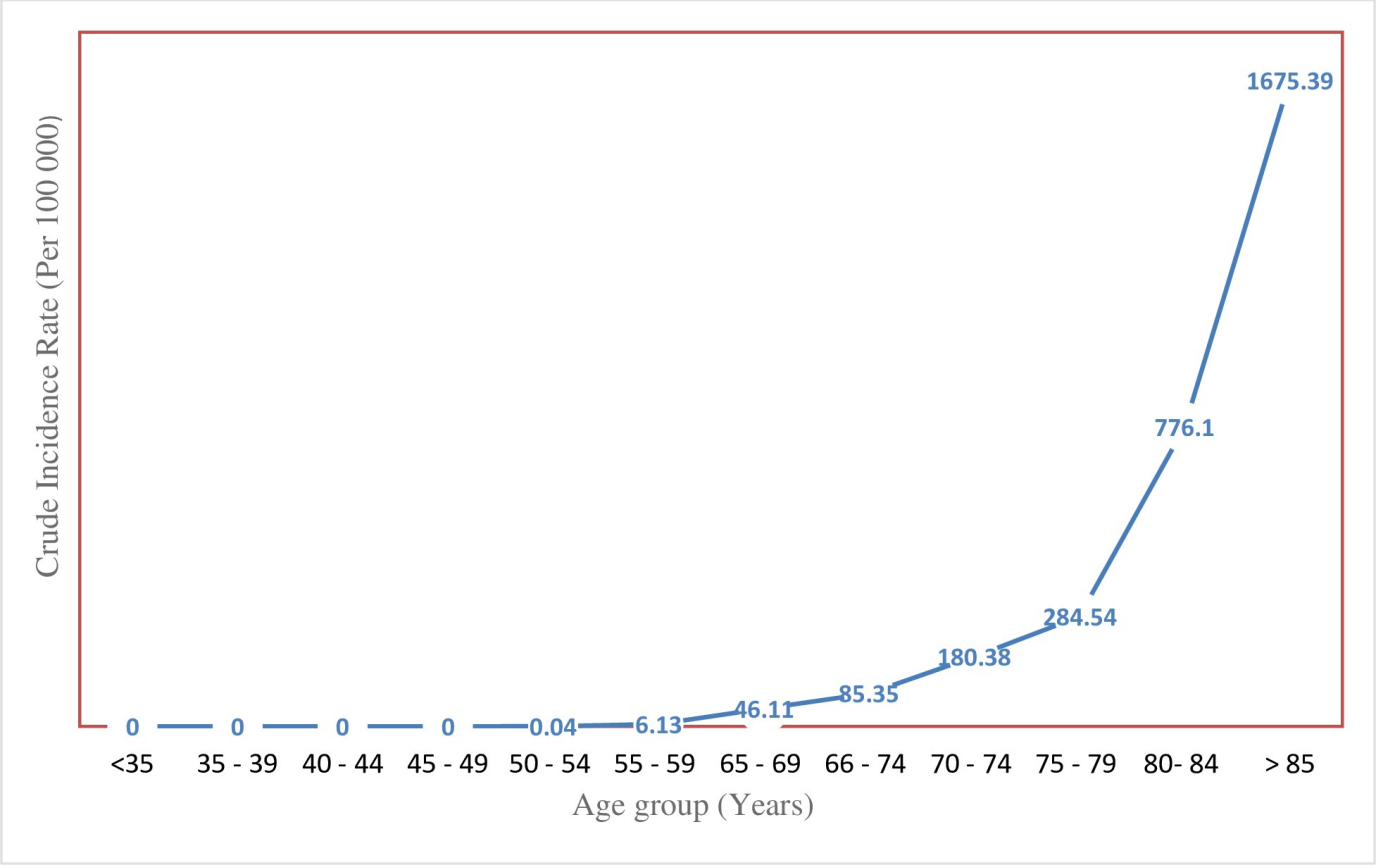
Age specific Crude incidence rates per 100 000 for prostate cancer between 2011–2018 by population group.

The Crude incidence rates per 100 000 persons for prostate cancer is shown in Fig 1. Analysis of age-specific crude incidence rates shows that PCa cases were: 0.04, ages < 55 years; 0.13, ages 55–59 years; 46.11 ages 60–64; 85.35, ages 65–69 years; 180.38 ages 70–74 years; 284.54 ages 75–79 years, 776.1 ages 80–84 years and 1675.39 ages ≥ 85 years.

The age-specific rates crude rates, and age-standardised rates for individual years are shown in Table 4. The AAPC (CI) was 44.7 (36.1–53.7), P value < 0.001 showing a strong association between age standardised rates and age bands. Only one joint segment was used in the analysis. In both cases, the age-standardised rates were smaller than crude rates. The age standardised incidence rates (ASIR) for the respective age bands were: 0.03, ages < 55 years; 0.28, ages 55–59 years; 1.85 ages 60–64; 2.25, ages 65–69 years; 3.99 ages 70–74 years; 4.32 ages 75–79 years, 7.06 ages 80–84 years and 10.64 ages ≥ 85 years.

**Table 4:** Age-specific, crude, and overall age-standardised rates of prostate cancer in Eritrea (overall age-standardised rates used the hypothetical world population net 2014 for Eritrea and World standard population) 2011–2018.

Age group (Years)	Prostate Cancer cases per age group (CIR)							
	< 55	55–59	60–64	65–69	70–74	75–79	– 84	>85
2011	0(0.0)	1(2.04)	1(2.7)	4(14.2)	4(26.7)	4(56.9)	2(77.6)	0(0.0)
2012	0(0.0)	1(2.04)	3(8.1)	2(7.1)	4(26.7)	0(0.0)	1(38.8)	2(209.4)
2013	1(0.04)	0(0.0)	4(10.9)	2(13.6)	2(8.1)	2(5.4)	3(5.4)	3(8.14)
2014	0(0.0)	0(0.0)	8(13.6)	6(28.5)	2(13.4)	1(14.2)	2(77.6)	0(0.0)
2015	0(0.0)	0(0.0)	0(0.0)	3(10.7)	5(33.4)	2(28.5)	3(116.4)	3(314.1)
2016	0(0.0)	1(2.0)	0(0.0)	2(7.1)	4(26.7)	4(56.9)	3(116.4)	2(209.4)
2017	0(0.0)	0(0.0)	0(0.0)	2(7.1)	2(13.4)	3(42.7)	4(155.2)	3(314.1)
2018	0(0.0)	0(0.0)	1(2.7)	3(10.7)	4(26.7)	4(56.9)	2(77.6)	3(314.1)
**ASIR (World)**	**0.03**	**0.28**	**1.85**	**2.25**	**3.99**	**4.32**	**7.06**	**10.64**

**ASIR**: Age standardised incidence rates; **CIR:** crude incidence rates.

The overall cases per year, MV%, CIR, standard errors and ASIR and APCs for 2011–2018 are shown in Table 5. The MV% during the period was 36.7%. The CIR and ASIR were 5 per 100 000 and 30.26 per 100 000 in 2011–2018. The average ASIR values over the study period was 3.78 per 100 000. The AAPCs during the same period was 5.4% (95% CI = −1.4, 12.7). See S1 Appendix.

**Table 5:** Eight-year age standardized incidence rates and crude incidence rates of prostate cancer (per 100 000 population) (2011–2018).

Years	Number of incident events (Cases)	MV%	Crude incidence rate (CIR)	Standard error	Age-adjusted incidence rate (ASIR) (World)
**2011**	16	68.8	0.63	0.16	2.70
**2012**	13	69.2	0.51	0.14	2.92
**2013**	17	5.9	0.66	0.16	4.50
**2014**	19	26.3	0.74	0.17	2.69
**2015**	16	12.5	0.63	0.16	4.46
**2016**	16	25.0	0.63	0.16	4.09
**2017**	14	78.6	0.55	0.15	4.51
**2018**	17	23.5	0.66	0.16	4.39
*P-value*	**0.100**				
**AAPC**	**5.4**				
**95% CI**	**(- 1.4, 12.7)**				
**Total**	**128**	**36.7**	**5 per 100000**		**30.26 per 100000**

**MV%**: Microscopically verified percentage, **AAPC**: Average annual percentage change.

## Discussion

Prostate cancer is a serious personal, and to some extent, public health problem affecting elderly populations worldwide. It is the 4^th^ most common cancer overall and the 2^nd^ most common cancer in men [5, 3]. As stated previously, cancer registration is an incredibly difficult pursuit in Africa. These has undermined the public availability of solid data on PCa prevalence, incidence and mortality from the region. This concern was adequately captured by Adeloye and colleague’s [5] assertion that there is relatively low level of research on PCa incidence in Africa. For a large proportion of countries in the region (Eritrea included), even low-quality population-based cancer registries (PBCR) are unavailable [12]. In fact, that only 1 in 3 PBCRs report high-quality cancer data to the International Agency for Research on Cancer (IARC) [3]. As a consequence, past GLOBOCAN country-specific estimates for Eritrea were modeled using MR/IRs derived from cancer registry data in neighboring countries [12]. Needless to say, there is much uncertainty associated with this approach. To address these uncertainties, we evaluated the incidence of PCa in Eritrea using data aggregated from three of the most reliable data repositories in the country.

In the present study, we demonstrated that Eritrea has a relatively static, although substantial incidence of PCa. The highest and lowest PCa incidence rates were in 2017 (4.51 per 100 000) and 2014 (2.69 per 100 000), respectively. The age standardised rates (ASIR) (World) over the study period (2011–2018) was 30.26 per 100 000. Therefore, annual ASIR values over the study period was 3.78 per 100 000. Overall, the current figures are higher than previous estimates for the country (ASIR 0.7, (Period: 2000–2010) [18]. In contrast, our figures are within the range reported by GLOBOCAN in their latest PCa estimate for Eritrea (≤ 16.3 per 100 000) [2]. It is also notable that these values are somewhat lower than the values for Eastern Africa reported in GLOBOCAN 2018: Addis Ababa, Ethiopia (Year: 2012–2013), 5.7 per 100 000; Eldoret, Kenya (Year: 2008−2011), 15.8 per 100 000; Nairobi, Kenya (Year: 2007−2011); 43.9 per 100 000; Kampala, Uganda (Year: 2008−2012), 49.5 per 100 000 [6]. The significant inter and intra-country variation in the incidence of PCa possibly reflects the multifactorial impacts of genetic variation; environmental factors; overall life expectancy; access to medical care; and variations in use of PSA-based screening protocols/or policies, among others [19, 20]. On a global scale, our data reinforces the widely reported fact that PCa incidence in native African populations is generally low vis-à-vis that of African-Americans, Afro-Caribbean’s and Black men in Europe [20–23]. As an example of the stark disparity in reported PCa incidence between native African men and African American men; the Gambia had an incidence rate of 4.7 per 100 000 during 1997–1998 versus an African American incidence rate of between 80–195.3 per 100 000 during the same period [23].

Based on the fact that the data used in this analysis was pooled from all the regions in the country (Table 1), it can be argued that its fairly representative–the marked differences in the number of cases submitted from the various sub-regions notwithstanding. Importantly, PCa incidence between and within countries often reflect significant disparities. Multiple observers have noted that cancer data from SSA are mostly sourced from urban centers and are gravely under—representative of rural areas [5]. Underlying causes of the observed phenomenon are multi-factorial and debatable [2, 10, 20, 21, 24]. For instance, the high proportion of PCa incidence in developed countries or urban Africa is generally attributed to the influence of screening (PSA testing of asymptomatic men) and ‘‘incidental” diagnosis (histological examination of prostatectomy specimens) [1,3]. Like most countries in Africa [6], very few prostatectomy specimens are referred for histological examination in Eritrea. Many patients also present with advanced or metastatic disease [5, 25]. To be sure, a majority of the patients in this study were diagnosed with advanced disease on the basis of clinical case ascertainment or case histories. In addition, most of the histologically diagnosed PCa cases had a Gleason Score ≥ 7).

As previously noted, differences in lifestyle, access to medical care and quality of registries maybe be responsible for the geographic and racial/ethnic disparities. Reliance on hospital-based rather than PBCR or population-based sampling and lack of pathologists/histopathologists have also been mentioned [5, 20, 21]. Based on these considerations, PCa incidence rates in Africa are projected to increase as early detection, improvements in clinical diagnosis protocols, access to medical care, better reporting, and improvements in documentation/ascertainment of cases [20]. These propositions may likely be true for Eritrea. Actually, it’s our opinion that multiple areas for improvements exists particularly for cancer research. In addition, no data exist regarding the prevalence of PSA usage in Eritrea and the relationship between detection rate of PCa and PSA. Data specifically targeted at capturing the comparative experiences and views of physicians or other health personnel on PSA usage or protocols for reference for DRE, or TRUS-guided biopsies are lacking. Information on medical belief/practices of populations at risk is also required. Research on risks factors (including genetic studies) is also evidenced by the evidence linking PCa and specific risk factors (increased body weight, obesogenic diet, physical inactivity, Diabetes mellitus (DM), among others [9, 22, 26, 27, 28] which are currently driving the on-going epidemiological transition in Africa.

In the age group analysis, we observed an increasing trend in PCa incidence with advancing age. The CIR for individuals over 70 years was relatively large (Table 3). In contrast, the data for the lower age bands were comparable to those from other parts of Africa [5, 26]. Clearly, the data corroborates the widely held assertion that PCa is one of the most age-dependent malignancies—rare before the age ≤ 50 years, it increases at an exponential rate, thereafter [26, 27]. On the basis of this observation, some scholars have noted that lower incidence of PCa in SSA may be related to the comparably lower longevity in some African countries. In other words, men do not live long enough in some of countries in Africa to develop or manifest signs of the disease [20]. This possibility has far reaching implication for countries like Eritrea where significant gains in life expectancy have been reported in the last decades–life expectancy at birth is 64.9 years, up from 36 years in 1990 [29, 30]. Therefore, understanding the impact of the changing age structure (e.g. aging-adjusted incidence rate) within this population and its relationship to PCa incidence is important [31]. Regardless, the PCa data, and to some extent, the BPH data presented in this study indicates that the burden of age-related urological complications in men presents a gathering and potentially disruptive challenge to the country’s health system.

### Limitations of the study

Here in this report, we collected the first-hand clinical and laboratory statistics on PCa cases reported between 2011–2018 in specific representative institutions in Eritrea. However, this study could have been constrained by a number of factors. A key challenge is the fact that only a small fraction of the cases were referred for histological verification/Pathology confirmation. Further, the number of cases from specific administrative zones (Zobas) was relatively small–(Southern Red Sea and Northern Red Sea). These administrative zones are sparsely populated and have some of the remotest regions in the country. Therefore, access to medical facilities, particularly to the centrally located referral facilities, may undermine the completeness of our data. Considering these region-based inequalities, the data may potentially underestimate the incidence of PCa in Eritrea. However, as reported earlier, these gaps, although expected, may only have a marginal effect. Lastly, unavailability of updated population data for estimation of incidence may also be an issue. Indeed, a separate sub-analysis of PCa burden in the disparate administrative zones was not attempted for the same reason.

## Conclusion

Estimating the true incidence of PCa in Africa presents several enduring challenges that continue to defy solution despite extensive research. The aims of this study were to contribute to improving the evidence on the burden of PCa in Eritrea by collecting data from fairly representative hospitals and laboratories in the country. In aggregate, we uncovered a relatively stable incidence (APCC = 5.4 95% CI—1.4, 12.7, p value– 0.100) with highest and lowest PCa incidence in 2017 (4.51 per 100 000) and 2014 (2.69 per 100 000), respectively. The overall ASIR between 2011–2018 was 30.26 per 100 000. In the age group analysis, we observed an increasing trend in PCa incidence with advancing age. Further, we uncovered marked heterogeneity in the absolute number of cases reported from the disparate administrative zones. Although the reasons for this phenomenon are likely to be multifaceted; the influence of differential access to screening, physicians and referral patterns may be prominent. Interestingly, the study appears to suggests that previous reports have under-estimated the incidence of PCa in Eritrea. Lastly, the study provides ample evidence on the need for research targeted at uncovering the true burden of PCa in Eritrea. Potential solutions will require the establishment of high-quality PBCRs and improvisation of research policies/or long-term commitment to improvements in research, training, screening, diagnosis, and the overall management of PCa in the country.

## Supporting information

S1 Rawdata (SAV)

S1 Appendix Joint point regression. (DOCX)

## Abbreviations

ASIR: Age standardised Incidence rates
CIR: Crude incidence rates
APC: Annual percentage change
PCa: Prostate Cancer
NHL: National Health Laboratory
PBCR: Population-based cancer registry
SSA: sub-Saharan Africa
DM: Diabetes Mellitus
IHME: Institute for Health Metrics and Evaluation
DALYs: Disability adjusted life years
PSA: Prostate specific antigen
NCRS: National Cancer Registration Systems
LMIC: Low- and medium-income countries
