# Supplementary figures and images for: Incidence of prostate cancer in Eritrea: Data from the National Health Laboratory, Orotta Referral Hospital and Sembel Hospital 2011-2018
Source: PLoS One. 2020 Apr 23;15(4):e0232091. doi: 10.1371/journal.pone.0232091 (PMC7179877; doi:10.1371/journal.pone.0232091)

**Appendix 1**: Joint Point Regression


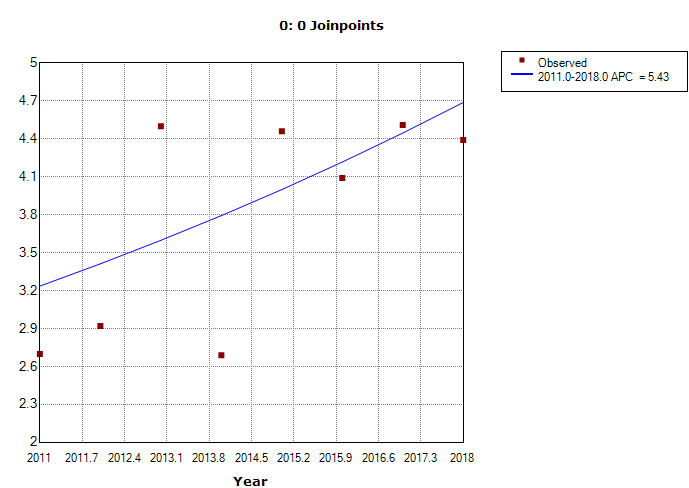

Supplement: S1 Appendix — (DOCX) [file pone.0232091.s002.docx]
